# RNA Interference Mitigates Motor and Neuropathological Deficits in a Cerebellar Mouse Model of Machado-Joseph Disease

**DOI:** 10.1371/journal.pone.0100086

**Published:** 2014-08-21

**Authors:** Clévio Nóbrega, Isabel Nascimento-Ferreira, Isabel Onofre, David Albuquerque, Nicole Déglon, Luís Pereira de Almeida

**Affiliations:** 1 CNC - Center for Neuroscience & Cell Biology, University of Coimbra, Coimbra, Portugal; 2 Faculty of Pharmacy, University of Coimbra, Coimbra, Portugal; 3 Faculty of Sciences and Technology, University of Coimbra, Coimbra, Portugal; 4 Lausanne University Hospital, Department of Clinical Neurosciences, Laboratory of Cellular and Molecular Neurotherapies, Lausanne, Switzerland; Northwestern University, United States of America

## Abstract

Machado-Joseph disease or Spinocerebellar ataxia type 3 is a progressive fatal neurodegenerative disorder caused by the polyglutamine-expanded protein ataxin-3. Recent studies demonstrate that RNA interference is a promising approach for the treatment of Machado-Joseph disease. However, whether gene silencing at an early time-point is able to prevent the appearance of motor behavior deficits typical of the disease when initiated before onset of the disease had not been explored. Here, using a lentiviral-mediated allele-specific silencing of mutant ataxin-3 in an early pre-symptomatic cerebellar mouse model of Machado-Joseph disease we show that this strategy hampers the development of the motor and neuropathological phenotypic characteristics of the disease. At the histological level, the RNA-specific silencing of mutant ataxin-3 decreased formation of mutant ataxin-3 aggregates, preserved Purkinje cell morphology and expression of neuronal markers while reducing cell death. Importantly, gene silencing prevented the development of impairments in balance, motor coordination, gait and hyperactivity observed in control mice. These data support the therapeutic potential of RNA interference for Machado-Joseph disease and constitute a proof of principle of the beneficial effects of early allele-specific silencing for therapy of this disease.

## Introduction

Machado-Joseph disease (MJD) or Spinocerebellar ataxia type 3 (SCA3) is a dominant fatal inherited disorder of the central nervous system (CNS) caused by overrepetition of the CAG trinucleotide in the coding region of the *MJD1/ATXN3* gene, which encodes the ataxin-3 protein (Atx3) [1]. In this work we characterize the effects of mutant ataxin-3 silencing on MJD-associated motor behavior and neuropathological abnormalities in a pre-symptomatic cerebellar mouse model by co-injecting lentiviral vectors encoding for the silencing short hairpins RNAs and vectors encoding for mutant ataxin-3. We show that early mutant ataxin-3 silencing abolishes the appearance of balance and motor coordination deficits, ataxic gait and histological hallmarks of the disease.

MJD belongs to a wide group of similar disorders designated polyglutamine diseases [2]. MJD is characterized by diverse clinical presentation, particularly cerebellar ataxia, along with other symptoms such as peripheral neuropathy, bulging eyes, ophthalmoplegia, dystonia, nystagmus and fasciculations [3]–[6]. A common hallmark of the disease is the accumulation of abnormally misfolded protein, typically in the form of intranuclear neuronal inclusions [7] in affected brain regions, such as afferent and efferent cerebellar systems, substantia nigra, cranial nerve motor nuclei [5] and striatum [8]–[11]. MJD is the most common ataxia worldwide [12] and similarly to most other polyglutamine diseases no treatment is available.

In the last years, the strategy of gene silencing by RNA interference (RNAi) has been proposed to knock-down the expression of mutant genes in order to rescue the phenotype of dominant disorders, including polyglutamine diseases [13]. Other strategies have been proposed to improve mutant protein clearance and mitigate its toxic effects [14]–[16], but within the molecular cascade that leads ultimately to neuronal dysfunction and cell death, none of these acts as early as RNA interference, which we and others have shown to be effective to treat several diseases including MJD [17]–[21]. However, for the specific case of MJD, *in vivo* studies testing the ability of gene silencing initiated at an early stage to prevent the appearance and progression of motor behavior abnormalities were missing. This is particularly relevant in MJD as patient's genotyping could allow initiation of treatment before the appearance of the first symptoms. The lentiviral mouse model here used is particularly suited for this specific study as it allows initiation of the knock-down of mutant ataxin-3 at an early time-point before onset of symptoms.

One important issue to consider is whether allele-specific silencing of mutant ataxin-3 or generalized silencing of both alleles of the protein, wild-type and expanded/mutant should be done. The ataxin-3 protein has a de-ubiquitinating enzyme activity [22], [23] and it has also been linked to aggresome formation [24], endoplasmatic reticulum-associated degradation [25], cytoskeleton network [26], among other roles. Therefore, although knock-out mice for ataxin-3 have no major abnormalities [27], [28] and generalized silencing of ataxin-3 in the context of MJD has proved to be safe and effective [29], here we used the more cautious approach of allele-specific silencing for the mutant ataxin-3. This strategy takes advantage of a single nucleotide polymorphism, which can discriminate the mutant allele in approximately 70% of MJD patients [17]–[21], [30].

We demonstrate therapeutic efficacy of the gene silencing approach in this pre-symptomatic cerebellar model in abolishing the appearance of the representative MJD gait, balance and motor coordination abnormalities, as well as neuropathology, supporting the beneficial effects of this strategy as a therapeutic approach for MJD.

## Results

### Allele-specific silencing of mutant ataxin-3 prevents motor coordination and gait deficits

**Figure 1:**
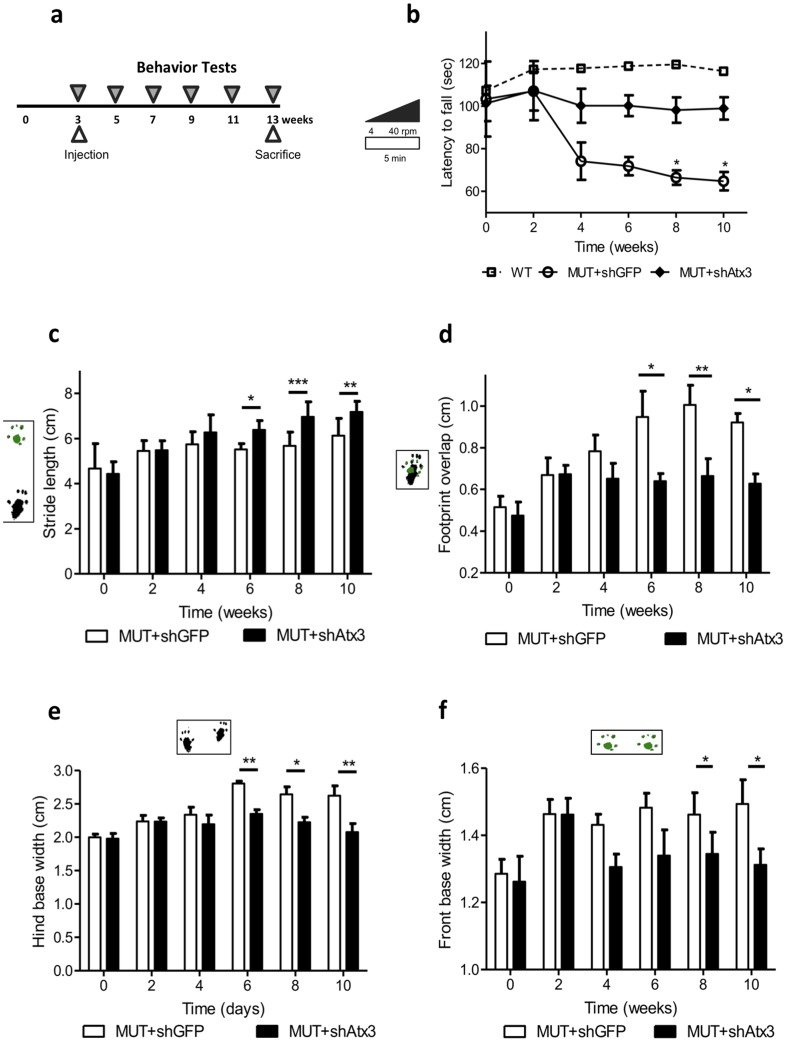
Allele specific silencing of mutant ataxin-3 prevents the appearance of balance and motor coordination abnormalities. (a) Time course of mice behavior tests: rotarod performance test, footprints patterns analysis and activity box monitoring. (b) Rotarod test. Mice were placed in a rotarod accelerating from 0 to 40 r.p.m. in 5 min. Mice co-injected with Atx3 MUT and shAtx3 (closed circles, *n* = 8) showed an improvement performance relative to mice co-injected with Atx3 MUT and shGFP (control) (open circles, *n* = 7), especially at 8 and 10 weeks post-injection. Wild-type non-injected mice are represented as WT (*n* = 8). Values are expressed as mean ± SEM. *P<0.05 (2-way ANOVA Bonferroni *post*-test). (c–f) Footprint analysis of shAtx3 and shGFP-treated Atx3 MUT mice. (c) Stride length. Mice injected with RNA interference against mutant ataxin-3 (MUT+shAtx3) have an increased stride length relative to control (MUT+shGFP) starting at 6 weeks post-injection. Values are expressed as mean ± SEM. *P<0.05, **P<0.01, ***P<0.001 (2-way ANOVA Bonferroni *post*-test). (d) Footprint overlap. The distance between the front and hind footprint placement or overlap is reduced in shAtx3-injected mice relative to control, starting at 6 weeks post-injection. Values are expressed as mean ± SEM. *P<0.05, **P<0.01 (2-way ANOVA Bonferroni *post*-test). (e) Hind base width. The shAtx3-injected mice have a narrow hind base width relative to control, starting at 6 weeks post-injection. Values are expressed as mean ± SEM. *P<0.05, **P<0.01 (2-way ANOVA Bonferroni *post*-test). (f) Front base width. The shAtx3-injected mice have a narrow front base width relative to control, starting at 8 weeks post-injection. Values are expressed as mean ± SEM. *P<0.05 (2-way ANOVA Bonferroni *post*-test). In general the patterns clearly differ, showing that shAtx3-injected mice display longer strides, evenly spaced and accurately positioned footprints when compared to control-treated MJD mice.

In this study, to explore the outcome of mutant ataxin-3 gene silencing in MJD motor phenotype and neuropathology, lentiviral vectors encoding RNA interference transcripts were co-injected with the human full-length mutant ataxin-3 in the cerebellum of wild-type mice (Figure S1 in File S1). To ensure the specific silencing of mutant ataxin-3, we used short-hairpin RNAs (shRNA) specifically targeting the mutant ataxin-3 allele (shAtx3, *n* = 8), carrying a previously characterized single nucleotide polymorphism [18]. A similar group of mice injected with a control shRNA sequence against green fluorescent protein (shGFP, *n* = 7) was used as control. To analyze whether mutant ataxin-3 silencing would prevent appearance of a MJD- typical motor phenotype [31], motor behavior was assessed every 2 weeks during 10 weeks post-injection (Fig. 1 a).

In the accelerating rotarod test (acceleration from 4 to 40 rpm in 5 min) (Fig. 1 b and Figure S2 in File S1), mice injected with shAtx3 (Fig. 1 b); closed diamonds, *n* = 8) exhibited a performance that was not significantly different from wild-type non-injected animals (open squares) at any time-point. In opposition mice injected with shGFP (control, Fig. 1 b, open circles, *n* = 7), displayed a significant loss of performance already at 4 weeks post-injection, being statistically different at longer time-points (Fig. 1 b; *n* = 7–8; T8 and T10: *P<0.05). In fact, while the control-treated MJD mice performance aggravated along time, shAtx3-treated mice had a constant and normal performance during the whole period of the test, and similar to wild-type non-injected mice performance (Figure S2 in File S1). These data suggest that gene silencing is able to hamper the appearance of balance and motor coordination abnormalities, common symptoms observed in MJD.

Another symptom frequently observed in MJD patients and MJD mouse models is the presence of gait ataxia [31]–[35]. To access if mutant ataxin-3 silencing was able to impede the development of an ataxic gait, footprint quantitative analysis was performed (Fig. 1 c–f). The footprint patterns revealed that shAtx3- injected mice had a significantly increased stride length relative to shGFP-injected mice starting at 6 weeks post-injection (Fig. 1 c; *n* = 7–8; T6: *P<0.05; T8: ***P<0.001; T10: **P<0.01). The distance between the front and hind footprint placement or footprint overlap was shorter in shAtx3 mice relative to shGFP, starting at 6 weeks post-injection (Fig. 1 d; *n* = 7–8; T6: *P<0.05; T8: **P<0.01; T10: *P<0.05), and the width between left and right footstep of hind (Fig. 1 e; *n* = 7–8; T6: **P<0.01; T8: *P<0.05; T10: **P<0.05) and front paws (Fig. 1 f; *n* = 7–8; T8: *P<0.05; T10: *P<0.05), showed that shAtx3-injected mice had a narrow hind and front base width relative to control (shGFP). The footprint patterns quantitative analysis revealed that shAtx3-injected mice revealed similar values to non-injected mice in all measurements [31]. Altogether this data show that mutant ataxin-3 gene silencing is efficient in preventing the appearance of an ataxic gait.

### Allele specific silencing reduces the hyperactivity observed in the MJD mouse model

**Figure 2:**
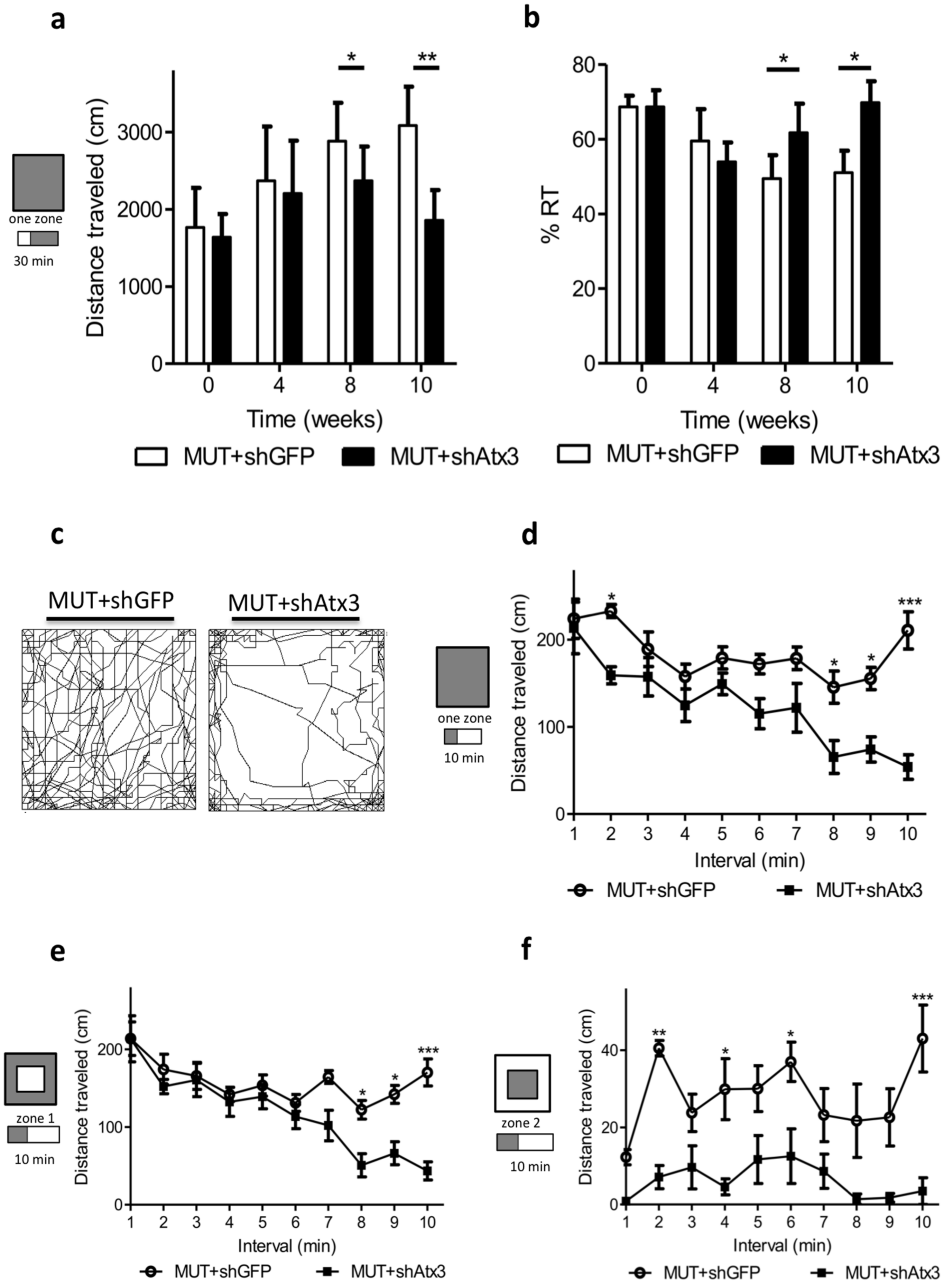
Allele specific silencing of mutant ataxin-3 reduces hyperactivity. (a, b) Analysis of the activity in the last 30 minutes (one zone). (a) Distance travelled. Mice injected with shAtx3 travelled less than control-injected mice starting at 8 weeks post-injection. Values are expressed as mean ± SEM. *P<0.05, ** *P*<0.001 (2-way ANOVA Bonferroni *post*-test). (b) Time spent on resting. Mice injected with shAtx3 spent more time resting than control-injected mice starting at 8 weeks post-injection. Values are expressed as mean ± SEM. *P<0.05 (2-way ANOVA Bonferroni *post*-test). (c) Plots of the moved track in the arena during the 40 minutes in the activity box at 10 weeks post-injection showed a clear difference between animals injected with shAtx3 and control mice. (d–f) Analysis minute-by-minute of distance travelled in the last time-point (10 weeks post-injection). (d) Distance travelled in the entire arena (one zone) during the first 10 minutes of the test. Values are expressed as mean ± SEM. *P<0.05, ***P<0.001 (2-way ANOVA Bonferroni *post*-test). (e) Analysis of the periphery of the arena (zone 1) during the first 10 minutes. This zone is associated with comfort and shelter. (f) Analysis of the center of the arena (zone 2) during the first 10 minutes. This zone is associated with reduced anxiety and fear. Mice injected with shAtx3 displayed a reduced hyperactivity relative to control, preferring the comfort zone (zone 1) relative to the center (zone 2). Taken together the significant differences were evident almost in all time points. Values are expressed as mean ± SEM. *P<0.05, **P<0.01, ***P<0.001 (2-way ANOVA Bonferroni *post*-test).

In view of these results we next investigated the activity pattern of RNA interference-injected MJD mice. Explorative behavior and general activity measurements were analyzed by the open field test before the injection, and 4, 8 and 10 weeks post-injection during 40 minutes per time point (Fig. 2). Allele-specific silencing of mutant ataxin-3 reduced the hyperactivity that characterizes mice upon overexpression of mutant ataxin-3 in the cerebellum [31]. Thus, these animals traveled less distance than control-injected animals (Fig. 2 a; *n* = 8; T8: *P<0.05; T10: **P<0.01), and spent more time resting (Fig. 2 b; *n* = 8; T8: *P<0.05; T10: *P<0.05), with a similar pattern of non-injected control mice [31]. In addition, by focusing the analysis in the last time point (Fig. 2 c–f, 10 weeks post-injection) we could observe that shAtx3-expressing mice travelled consistently less than shGFP mice during the whole 40 minutes of the test (Fig. 2 c). This difference was already present in the first 10 minutes of the analysis (Fig. 2 d; *n* = 7; T2: *P<0.05; T8: *P<0.05; T9: *P<0.05; T10: ***P<0.001), correlating with a decrease in the explorative behavior of the shAtx3-expressing mice. Furthermore, by dividing the analysis of this first 10 minutes in 2 different arena zones, periphery/zone1 and center/zone 2, interesting results were obtained (Fig. 2 e and f). The periphery of the arena is a comfort zone, correlating with shelter, while the center of the arena is an unprotected zone, correlating with reduced anxiety and fear [36], [37]. Mice expressing shAtx3 travelled less in the periphery (Fig. 2 e; *n* = 8; T8: *P<0.05; T9: *P<0.05; T10: ***P<0.001) and in the center (Fig. 2 f; *n* = 8; T2: **P<0.01; T4: *P<0.05; T6: *P<0.05; T10: ***P<0.001; Figure S3 in File S2), as compared to control mice. Interestingly, larger differences were observed in the center of the arena, with shAtx3 mice travelling substantially less in this zone when compared to the periphery. Overall these results suggest that control mice displayed a hyperactive phenotype associated with a reduced anxiety as compared to non-injected wild-type mice [31], which was prevented upon mutant ataxin-3 silencing.

### Expression of short-hairpin RNAs targeting mutant ataxin-3 in the cerebellum of a lentiviral-based MJD mouse model decreases the formation of intranuclear inclusions

**Figure 3:**
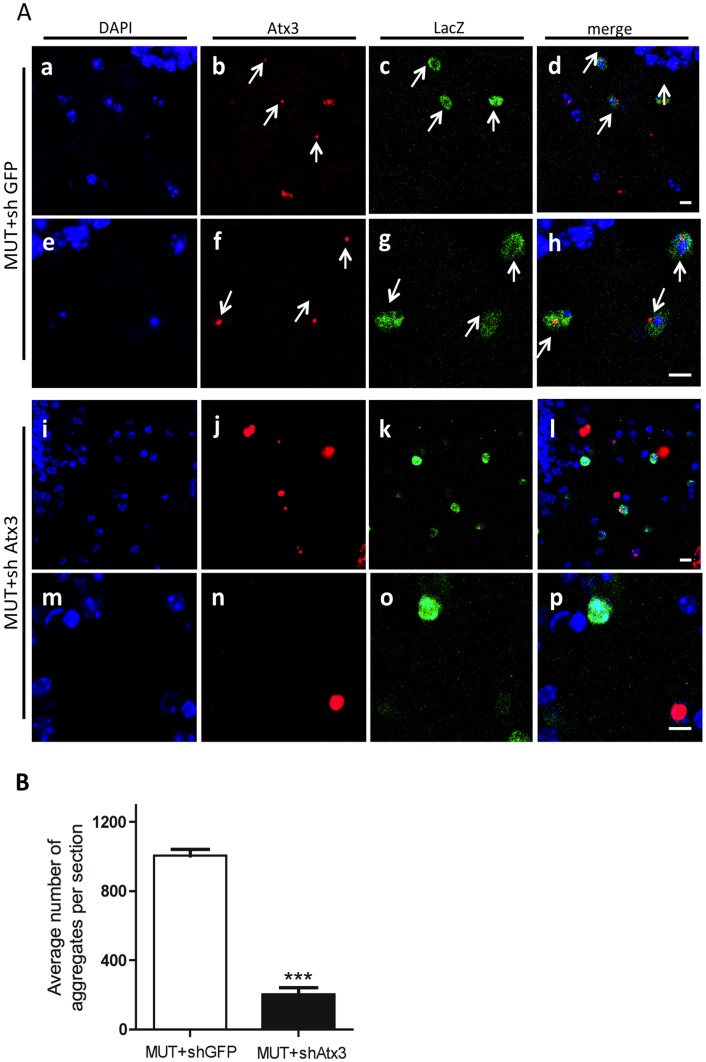
Allele-specific silencing of mutant ataxin-3 leads to diminution of the number of intranuclear aggregates. Confocal microscopy of ataxin-3, β-galactosidase, with nuclei counterstained in blue (DAPI). Mice injected with mutant ataxin-3 and short-hairpin against GFP (upper panel, MUT+ shGFP) exhibit aggregates of mutant ataxin-3 ((b,f), red, arrows) co-localizing with the short-hairpin RNA ((c,g), green, arrows); whereas aggregates from mice injected with mutant ataxin-3 and short-hairpin against mutant ataxin-3 (lower panel, MUT+ shAtx3) practically do not co-localize(l,p). This indicates that cells expressing the shRNA against mutant ataxin-3 do not display aggregates. Scale bar: 10 µm. (Q) Quantification of the mean number of aggregates per section. Silencing of mutant ataxin-3 significantly decreased the presence of mutant ataxin-3 aggregates (MUT+shAtx3) as compared to control (MUT+shGFP). Values are represented as mean ± SEM. *Statistical significance (*n* = 6; ***P<0.001; Unpaired Student's *t*-test).

An important hallmark of MJD is the presence of intranuclear aggregates or inclusions in the affected areas [7]. Histological analysis of the brain showed the presence of intranuclear mutant ataxin-3 inclusions in both groups, mainly present in the molecular layer of the cerebellar cortex (Fig. 3; Figure S4 in File S2). Nevertheless, while shGFP-expressing cells (Fig. 3A: a–h), revealed by lacZ reporter gene (Fig. 3A: c and g), also exhibited mutant ataxin-3 (Fig. 3A: b and f); shAtx3-expressing cells (Fig. 3A: i–p) did not stain for mutant ataxin-3 protein (Fig. 3A: j and n) in cells transduced with the ataxin-3-targeting shRNAs, and therefore positive for the lacZ reporter gene (Fig. 3A: k and o). This observation was further confirmed by quantitative analysis of the mean number of aggregates present in both groups, which revealed a significant reduction in the number of cells presenting mutant ataxin-3 aggregates in shAtx3-injected mice as compared to controls (Fig. 3B; *n* = 6; 202.9±40.56 versus 1004±37.77 in control; ***P<0.001).

**Figure 4:**
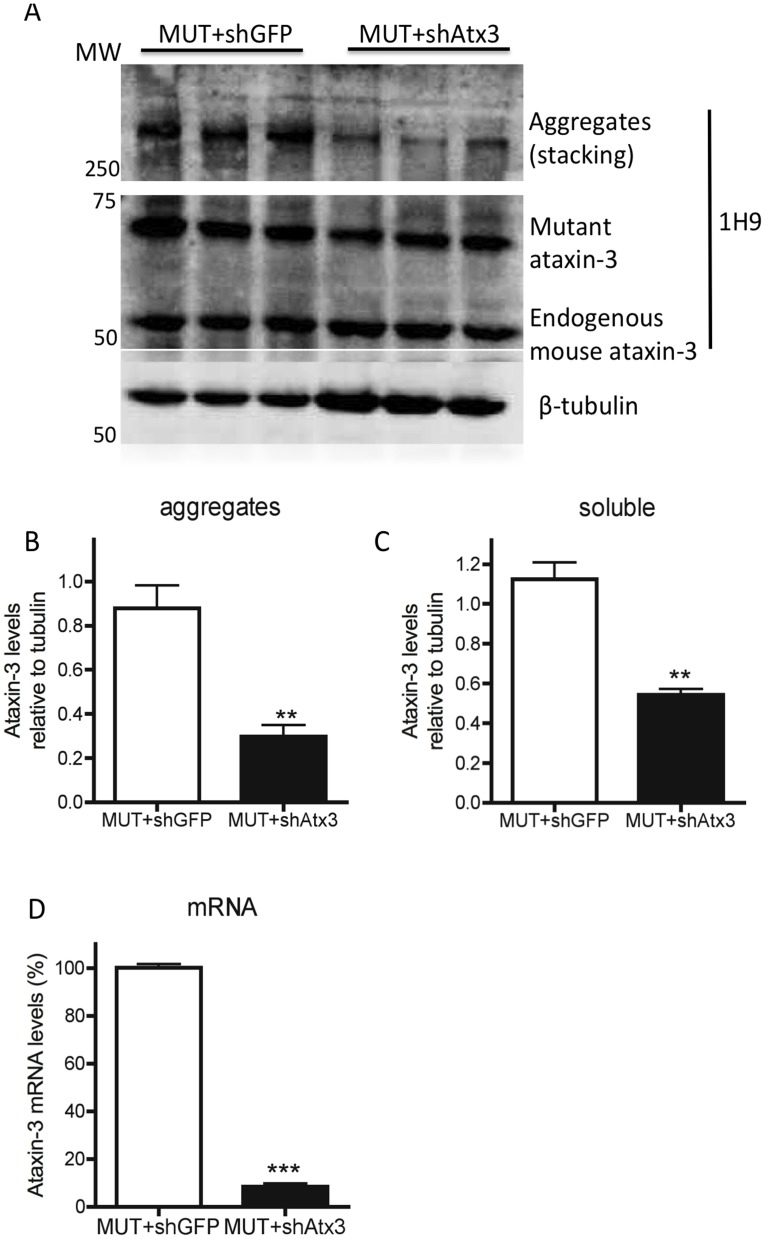
Allele-specific silencing of mutant ataxin-3 reduced the levels of mutant protein and mRNA. (A) Western blot analysis of cerebellar lysates stained with 1H9 antibody for ataxin-3. (B–D) Differences in the levels of high molecular weight protein species were detected between animals co-injected with MUT+shAtx3 (*n* = 3) and animals control (co-injected with MUT+shGFP, *n* = 3), whereas no differences were found in ataxin-3 endogenous levels. Normalization of protein levels was made with β-tubulin protein endogenous levels. (E) Quantitative real-time PCR analysis of cerebelar lysates shows a reduction in the ataxin-3 mRNA levels in the animals co-injected with MUR+shAtx3 compared to controls. Endogenous *hprt* mRNA was used as an internal control for the normalization and quantitative analysis of the ataxin-3 mRNA levels. Values are represented as mean ± SEM. *Statistical significance (*n* = 3; **P<0.01; ***P<0.001; Unpaired Student's *t*-test).

We further investigated the effects of mutant ataxin-3 silencing by western blot analysis of protein and real-time PCR of mRNA levels, in the mice cerebella (Fig. 4A). Probing the membrane with the 1H9 antibody, a marker for ataxin-3, revealed a robust and significant decrease in the levels of soluble (52%) and aggregated (66%) mutant ataxin-3 in the animals co-injected with shAtx3 compared to controls co-injected with shGFP (*n* = 3; aggregates at the upper level of the running and stacking gels Fig. 4B : 0.297±0.05 versus 0.878±0.10 in control, *P* = 0.0073, normalizing with tubulin; and soluble protein levels Fig. 4C : 0.543±0.02 versus 1.125±0.08 in control, *P* = 0.0027, normalizing with tubulin), whereas no difference was found between groups in the endogenous ataxin-3 levels (not shown). Moreover, the levels of ataxin-3 mRNA (Fig. 4D) were also strongly reduced in the animals co-injected with shAtx3 compared to control ones (*n* = 3; Fig. 4D: 8.432±1.255 versus 100±1.528 in control, *P*<0.0001, normalizing with hprt endogenous mRNA levels).

### Silencing of mutant ataxin-3 mediates neuroprotection to Purkinje cells

A common hallmark of polyglutamine diseases, including MJD is the loss of neuronal markers expression in affected regions [11], [32], [38]. Therefore, we next evaluated if the RNA interference treatment would be neuroprotective regarding mutant ataxin-3-associated loss of neuronal markers.

**Figure 5:**
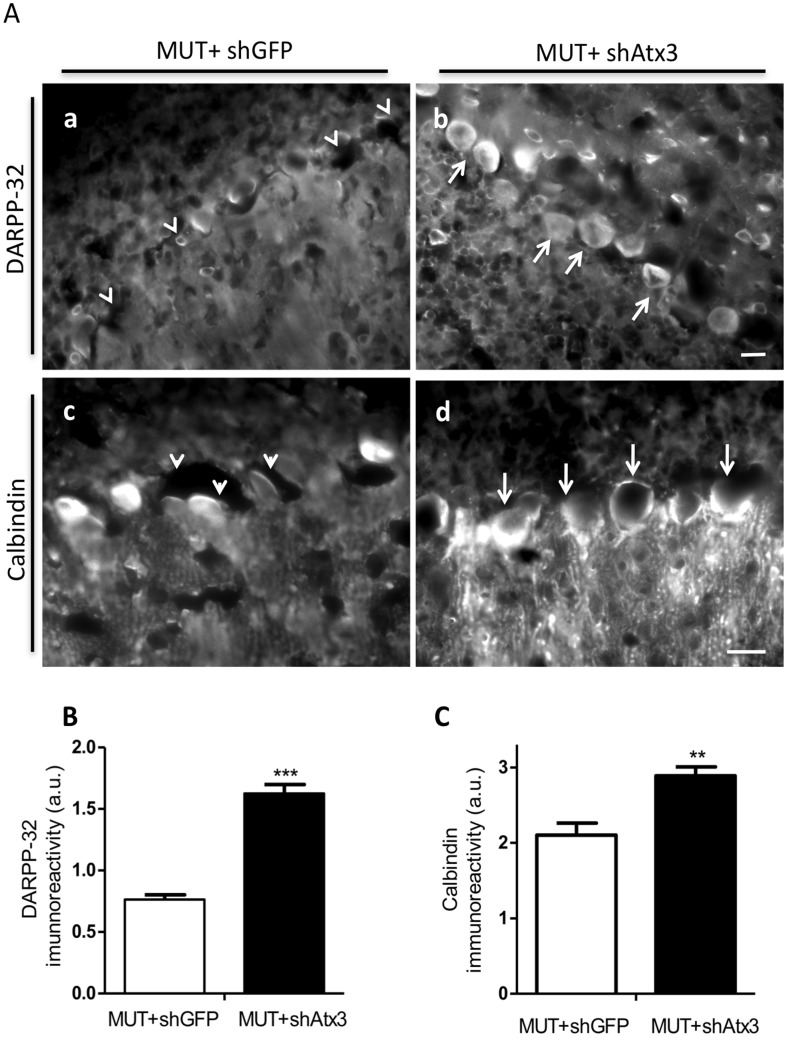
Allele-specific silencing prevents Purkinje cells pathology. Fluorescence microscopy analysis for the DARPP-32 (a,b) and calbindin (c,d) proteins highlighting the Purkinje cells. An increased expression of DARPP-32 neuronal marker was observed for the shAtx3-treated mice, relative to control-treated. Note the improved Purkinje cell morphology in shAtx3-treated mice (arrows) as compared to shrunken-sized Purkinje cells in control-treated (arrow heads). Scale bar: 40 µm. (E) Quantification of optical densitometry of DARPP-32 immunoreactivity. Silencing of mutant ataxin-3 significantly increased DARPP-32 expression (MUT+shAtx3) as compared to control (MUT+ shGFP). *Statistical significance (*n* = 5; ***P<0.001; Unpaired Student's *t*-test). (f–i) Fluorescence microscopy analysis for the calbindin protein highlighting the Purkinje cells and the molecular layer (c,d). An increased expression of calbindin neuronal marker was observed for the shAtx3-treated mice (d), relative to control-treated (c). Note the improved Purkinje cell morphology in shAtx3-treated mice and increased expression of calbindin in the molecular layer as compared to control. Scale bar: 40 µm. (F) Quantification of optical densitometry of calbindin immunoreactivity. *Statistical significance (*n* = 5; **P<0.01; Unpaired Student's *t*-test).

Fluorescent analysis of dopamine- and cyclic AMP-regulated phosphoprotein of molecular weight 32 KDa (Fig. 5A, upper panel: a–b) revealed that shAtx3-expressing mice displayed Purkinje cells with a normal morphology and immunoreactivity for DARPP-32 (arrows) while in control-treated mice Purkinje cells were shrunken and faintly immunoreactive to the DARPP-32 antibody (arrow heads). Quantitative analysis of the DARPP-32 immunoreactivity showed a preservation of DARPP-32 immunoreactivity in cerebellar sections of shAtx3-injected mice as compared to controls (Fig. 5 B; *n* = 5; 1.63±0.074 versus 0.76±0.038 in control; ***P<0.001). Analysis of the calbindin D-28K staining (Fig. 5A, lower panel: c–d) revealed a much stronger immunoreactivity for calbindin in Purkinje cells of shAtx3-expressing mice as compared to controls. This observation was further confirmed by quantitative analysis (Fig. 5C; *n* = 5; 2.889±0.1182 versus 2.103±0.1591 in control; **P<0.01). These data indicate that silencing of mutant ataxin-3 expression blocks the progression of neuronal dysfunction.

**Figure 6:**
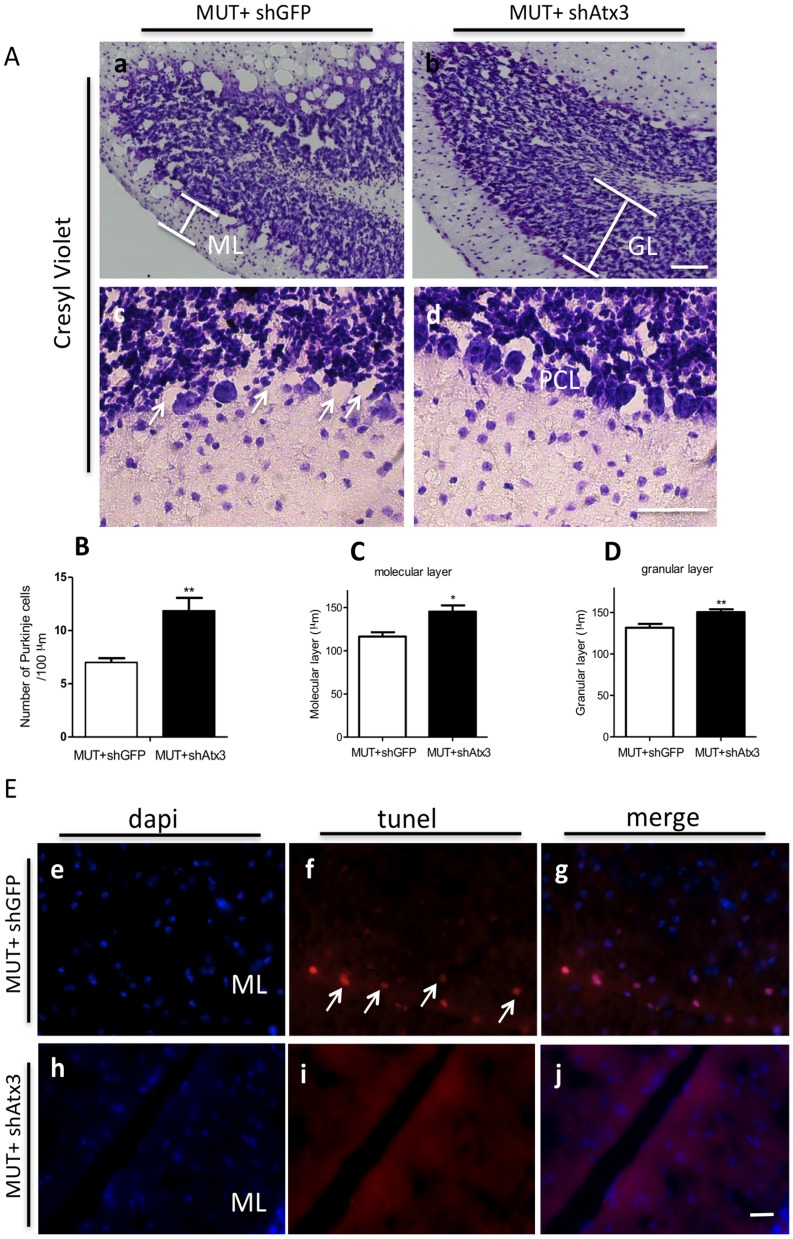
Silencing of mutant ataxin-3 reduces neurodegeneration. Cresyl violet staining. Note the increased cell number and improved cell morphology in shAtx3-treated mice (a–d). Scale bar: 20 µm. (E) Quantification of Purkinje cell number per µm. Silencing of mutant ataxin-3 significantly increased the cell number as compared to control. *Statistical significance (*n* = 4; **P<0.01; Unpaired Student's *t*-test). Moreover, the quantification of molecular (F) and granular layer (DG thickness also revealed a cellular preservation in mice injected with shAtx3 (*n* = 5; *P<0.05; **P<0.01; Unpaired Student's *t*-test, respectively). Apoptotic effect was detected in neurons (white arrows) in the control animals detected by TUNEL assay (red channel) (h–j) compared to mice co-injected with MUT+shAtx3 where no apoptotic effect was detected (k–m). ML, molecular layer; PCL, Purkinje cell layer; GL, granular layer.

To access if RNA interference-treatment was able to impede neurodegeneration and cell death, analysis of cresyl violet (Fig. 6A) and tunnel staining (Fig. 6E) was performed. Mice expressing shAtx3 revealed to have a significant increased number of Purkinje cells and overall cellular integrity as compared to control treated (Fig. 6A: a–d), which was further confirmed by quantitative analysis (Fig. 6B; *n* = 4; 11.85±1.22 versus 7.00±0.41 in control; **P<0.01). Moreover there was also a preservation of molecular layer thickness (Fig. 6C; *n* = 5; 145.1±7.21 versus 116.3±5.02 in control; *P<0.05) as well as of the granular layer thickness (Fig. 6D; *n* = 5; 150.3±2.69 versus 134.1±1.80; **P<0.01) in mice injected with shAtx3 compared to controls.

Consistent with these results, the Tunel assay also allowed detection of apoptotic cells in transduced areas of the molecular layer, in the control animals co-injected with shGFP in opposition to animals co- injected with shAtx3, which did not present tunel positive cells (Fig. 6E: e–j). Furthermore, FluoroJade-B staining revealed a consistently higher fluorescence consistent with neuronal degeneration in the molecular layer of the cerebellum in the control animals co-injected with shGFP (Figure S5 in File S2). Finally, Golgi staining revealed loss of dendritic arborizations in control animals compared to animals injected with shAtx3 (Figure S5 in File S2). Overall these data show that mutant ataxin-3 silencing prevents the appearance of the neurodegenerative pattern that is typical of this MJD model.

## Discussion

In this study, we investigated whether allele-specific gene silencing initiated before onset of symptoms would alleviate MJD. For this purpose we used an experimental paradigm involving simultaneous injection of lentiviral vectors encoding for the mutant ataxin-3 and for the shRNA sequences in the mouse cerebella, in this way mimicking a gene silencing therapy initiated at a pre-symptomatic stage. We found that this strategy is able to suppress or drastically reduce the development of motor impairments and neuropathological abnormalities representative of MJD.

The RNAi strategy has been proposed as potential therapy to down-regulate the expression of mutant genes and halt the progression of different autosomal dominant neurodegenerative diseases. To date its efficacy has been proved in several pre-clinical rodent trials for diseases such as Huntington's disease (HD) [39], [40], familial forms of amyotrophic lateral sclerosis (ALS) [41], [42] and spinocerebellar ataxia type 1 (SCA1) [43] as well as in familial forms of Alzheimer disease (AD) [44]. Among the important concerns relative to RNAi therapy is the development of allele-specific approaches in order to selectively target the mutant allele without inhibiting the corresponding wild-type allele. This concern is of particular importance in diseases where the knockdown of wild-type allele has proved to be toxic; nevertheless even when an obvious toxicity is not present, given the potential unknown side effects of long-term silencing of wild-type proteins, selective strategies offer a better therapeutic solution [45].

In previous work, we and others have shown that both allele-specific [18], [19] and undiscriminating silencing [20], [21], [29] were effective strategies for alleviating striatal neuropathology of MJD. Here we show that early allele-specific silencing of mutant ataxin-3 is able not only to impede the development of mutant ataxin-3 aggregated and associated neuronal dysfunction within the cerebellum but also to robustly prevent the progression of balance and motor coordination deficits measured by the accelerated rotarod test as well as gait analysis of footprints. Despite the limitations of the experimental paradigm, as simultaneous injection of vectors encoding for mutant ataxin-3 and for the silencing sequences (shAtx3) may prevent the levels of mutant ataxin-3 from reaching the levels found in controls, our data constitutes a proof-of-principle for initiation of therapy before onset of the disease.

In MJD patients, as well as other SCAs patients, the gait ataxia is a clinical symptom always present. Indeed, gait difficulty is reported as the initial symptom in 66% of the patients while for others, symptoms like cramps (9%), sleep disturbances (5%), neuropathic symptoms (3%), restless legs syndrome (3%) among others appear first [34]. In this study the allele-specific silencing was able to hamper the development and progression of gait ataxia, as measured by the specific test of footprint analysis.

Furthermore, gene silencing mediated alleviation of the hyperactive phenotype observed in this mouse model. Hyperactivity had been already reported for a mouse model of Dentatorubral-Pallidoluysian Atrophy (DRPLA) [46] and MJD [47]. This phenotype can be an indication of reduced emotionality [48] as observed in MJD patients [49]. However, other MJD mouse models presented hypoactivity [32], [50], [51]. In fact, this phenomenon can also be related to the restless legs syndrome and REM sleep behavior disorder, which are more frequent in MJD than other SCA patients (50%) [52], [53].

Regarding neuropathology, mutant ataxin-3 silencing was also able to reduce the mutant ataxin-3 aggregate formation and to hamper the development of Purkinje cells abnormalities and dysfunctional expression of neuronal markers, as observed in mice treated with control sequences (shGFP). These neuropathological findings correlate with the observed motor behavior effects and with the previous results obtained with this strategy [18], [19].

In summary, we show that early and permanent lentiviral-mediated expression of RNAi sequences (shRNAs) targeting mutant ataxin-3 can dramatically hamper the development of MJD-associated abnormalities, including behavioral and neuropathological deficits in a mouse model of MJD. Notwithstanding the limitations of the experimental paradigm these data suggest that early gene silencing prevents or delays disease progression. Because genetic testing can be performed as early as *in uterus*, this strategy might be of benefit in the future, and thus may constitute a therapeutic strategy for MJD.

## Materials and Methods

### Lentiviral vectors

Viral vectors encoding for short-hairpin RNAs against GFP (shGFP) and human mutant ataxin-3 (shAtx3), as well as human full length mutant ataxin-3 with 72 glutamines (Atx3 MUT/MUT) [18], were produced in human embryonic kidney (HEK) 293T cells using as four-plasmid system described previously [38]. The lentiviral particles produced were resuspended in phosphate-buffered saline (PBS) with 0.5% bovine serum albumin (BSA) and samples were matched for particle concentration by measuring HIV-1 p24 antigen content (RETROtek, Gentaur, Belgium). Viral stocks were stored at −80°C until use.

### *In vivo* experiments

#### Animals

Mice C57/BL6 were housed in a temperature-controlled room and maintained on a 12 h light/dark cycle. Food and water were available *ad libitum*. Research with animals was conducted by trained researchers in an approved animal facility under protocol approved by ORBEA (orgão responsável pelo Bem-estar Animal). The experiments were carried out in accordance with the European Community Council directive (86/609/EEC) for the care and use of laboratory animals.

#### Stereotaxic surgery

Mice were anesthetized by intraperitoneal injection of a mixture of ketamine (100 mg/kg) with xylazine (10 mg/kg). Concentrated lentiviral stocks were thawed on ice and resuspended by vortexing and particle content matched to 250'000 ng of p24/ml. Wild-type C57BL/6 mice received a single injection of 6 µl of Atx3 MUT+ shGFP (*n* = 7; 1∶1) and Atx3 MUT+ shAtx3 (*n* = 8; 1∶1) at a rate of 0.25 µl/min by means of an automatic injector (Stoelting Co., Wood Dale, IL, USA), at the following coordinates: −1.6 mm rostral to lambda, 0.0 mm midline, and −1.0 mm ventral to the skull surface, with the mouth bar set at −3.3 [31]. After the injection, the syringe needle was left in place for an additional 5 min to minimize backflow.

### Motor behavior tests

Mice were trained on a battery of motor tests starting at P21–25 and performed every 2 weeks until 10 weeks. All tests were performed in a dark room after 30 minutes of acclimatization.

#### Rotarod

The rotarod apparatus (Letica Scientific Instruments, Panlab, Barcelona, Spain) was used to measure fore and hind limb motor coordination and balance. Each mouse was placed on the rotarod at a constant speed (5 rpm) for a maximum of 5 min, and the latency to fall was recorded. Mice received four trials per time point. After this test each mouse was placed again on the rotarod but this time at an accelerated speed (4 to 40 rpm in 5 min) for a maximum of 5 min, and the latency to fall was recorded. Mice received four trials per time point. For analysis, the mean latency to fall off the rotarod of 3–4 trials was used.

#### Footprint test

The footprint test was used to compare the gait of shAtx3 and control- treated lentiviral MJD mice. To obtain the footprints, the hind- and forefeet of the mice were coated with black and green nontoxic paints, respectively. The animals were then allowed to walk along a 100-cm-long, 10-cm-wide runaway (with 15-cm high walls). A fresh sheet of white paper was placed on the floor of the runaway for each mouse run. The footprint patterns were analyzed for four step parameters (all measured in centimeters). (1) Stride length was measured as the average distance of forward movement between each stride. (2) Hind-base width and (3) front-base width were measured as the average distance between left and right hind footprints, respectively. These values were determined by measuring the perpendicular distance of a given step to a line connecting its opposite preceding and proceeding steps. (4) Distance from left or right front footprint/hind footprint overlap was used to measure uniformity of step alternation. When the center of the hind footprint fell on top of the center of the preceding front footprint, a value of zero was recorded. When the footprints did not overlap, the distance between the center of the footprints was recorded. A sequence of six consecutive steps was chosen for evaluation, excluding footprints made at the beginning and end of the run where the animal was initiating and finishing movement, respectively. The same operator made all footprints measurements blindly. The mean value of each set of 5–6 values was used for analysis.

#### Open field analysis

For the assessment of the explorative and general activity behavior, open field tests were performed. Mice were placed in a 50×50 cm arena with 50 cm high walls and their movement activity was recorded for 40 min using the Acti-Track System (Panlab, Barcelona, Spain). In the analysis, the first 10 min correspond to the exploratory behavior, the last 30 min to the general activity of the mice, the periphery of the arena a comfort and shelter zone and the center of the arena an insecure zone.

### Histological processing

#### Tissue preparation

Animals were killed by sodium pentobarbital overdose, transcardially perfused with a 4% paraformaldehyde fixative solution (PFA 4%, Fluka, Sigma, St. Louis, USA) followed by brain removal. After a cryoprotective incubation in 25% sucrose in 0.1 M PBS for 48 h, brains were frozen in dry ice (−80°C) and 20 µm coronal sections were cut at a cryostat-microtome (Leica CM3050S, Leica Microsystems Nussloch, Germany). Slices throughout the entire cerebellum were collected in *superfrost* plus microscope slides (Thermo Fisher Scientific, U.S.A.) and stored at −20°C before immunohistochemical processing.

#### Immunohistochemical procedure

The immunohistochemical procedure initiated with a 30 min dehydratation at 37°C followed by a 30 min hydration in 0.1 PBS and 1 h blocking in a 0.3% triton in 0.1 PBS with 10% normal goat serum both at room temperature (RT). The following primary antibodies diluted in a blocking solution with 0.1% triton were used: mouse monoclonal anti-ataxin-3 (1H9, Chemicon, Temecula, CA, USA; 1∶5000; 48 h, 4°C), rabbit polyclonal anti-β-galactosidase (Molecular Probes, Invitrogen; 1∶1000; 48 h, 4°C), rabbit polyclonal anti-DARPP-32 (Chemicon,Temecula, CA, USA; 1∶5000, 48 h, 4°C), rabbit polyclonal anti-calbindin D-28K (Chemicon,Temecula, CA, USA; 1∶1000, 48 h, 4°C). Sections were then incubated in secondary antibody, goat-anti rabbit and/or mouse conjugated to alexa 488 or 594 (Invitrogen) for 2 h/RT e and then mounted in mowiol (Sigma) with 4′,6′-diamidino-2-phenylindole.Fluorescence images were acquired with a Zeiss Axiovert 200 imaging microscope or LSM Zeiss microscope for double staining experiments.

#### Western blot

Mice cerebella were removed after a sodium pentobarbital overdose, and incubated on ice in a radioimmunoprecipitation assay-buffer solution (50 mM Tris HCl, pH 8, 150 nM NaCl, 1% NP-40, 0.5% sodium deoxycholate, 0.1% sodium dodecyl sulphate) containing proteases inhibitors (Roche diagnostics GmbH) followed by a 4 sec ultra-sound pulse (1 pulse/sec). Total protein lysates were stored at −80°C, protein concentration was determined with the Bradford protein assay (BioRad), and 30 µg of protein extract was resolved in sodium dodecyl sulphate-polyacrylamide gels (4% stacking and 10% running). The proteins were transferred onto polyvinylidene difluoride membranes (GE Healthcare) according to standard protocols. The immunobloting procedure was performed as described previously [11] with the respective primary antibody (1H9, Chemicon, Temecula, CA, USA; 1∶5000), followed by incubation wit the corresponding alkaline phosphatase-linked secondary antibody. Bands were visualized with Enhanced Chemifluorescence substrate (ECF, GE Healthcare) and chemifluorescence imaging (VersaDoc Imaging System Model 3000, Bio-Rad). Membranes were stripped using 0.1 M glycine pH 2.3 (30 min, room temperature) and reprobed with mouse monoclonal anti-β-actin antibody (1∶5000, Sigma). Densitometric analysis was carried out in the same gel using Image J software (NIH, USA).

#### RT-PCR analysis

Mice cerebella were removed after a sodium pentobarbital overdose, and the cerebelar cortex was stored at −80°C for posterior RNA extraction. Total RNA was extracted using the RNeasy Mini Kit following the manufacturer's instructions (Qiagen, CA, USA). Real-time quantitative RT-PCR was performed in duplicate for each sample with 0.4% random-primed cDNAs generated from 400 ng total RNA. PCR was carried out in a 15 µl reaction volume containing SYBR Green Fast (Bio-Rad), and 100 nM of QuantiTect Primer Assay for human ataxin-3 (QT00094927, Qiagen, CA, USA). An Applied Biosystems, Step One Plus thermal cycler was programmed for an initial denaturation step (95°C, 30 sec) followed by 45 amplification cycles (95°C, 5 sec; 55°C, 15 sec). The amplification rate for each target was evaluated from the cycle threshold (Ct) numbers obtained with cDNA dilutions, with corrections for mouse hprt levels (QuantiTect Primer Assay, QT00166768; Qiagen, CA, USA), which were assumed to be constant. Differences between control and experimental samples were calculated using the 22DDCt method [54].

#### Quantification of nuclear inclusions and neuronal markers expression

Quantification of ataxin-3 positive inclusions was performed blindly by scanning 4 coronal sections spread over the anterior-posterior extent of the cerebellum (inter-section distance: 240 µm), using a 20× objective on a Zeiss Axiovert 200 imaging microscope and an image acquisition and analysis software (ImageJ, NIH). For each coronal section and animal 8 fields were acquired, covering the same areas. The average number of inclusions in the cerebellum was calculated for each animal. The quantification of DARPP-32 and calbindin neuronal expression was performed blindly by scanning 4 coronal sections spread over the anterior-posterior extent of the cerebellum expressing the mutant ataxin-3 protein (inter-section distance: 240 µm), using a 40× and 20× objective on a Zeiss Axiovert 200 imaging microscope. For each coronal section and animal, 8 fields were acquired using the same exposure and covering the same areas. Optical densitometry analysis was performed using Image analysis software (ImageJ, National Intistute of Health, U.S.A). Cerebellar regions expressing DARPP-32/calbindin revealed to have an increased optical density value when compared to regions not expressing the protein. Values are represented as the mean value of DARPP-32/Calbindin optical density per section ± SEM (arbitrary units).

#### Cresyl violet staining

Coronal brain sections were stained with cresyl violet dye for 2 minutes, differentiated in acetate buffer pH 3.8 (2.72% sodium acetate and 1.2% acetic acid; 1∶4 v/v), dehydrated by passing twice through ethanol and toluol solutions, and mounted with Eukitt® (O. Kindler GmbH & CO. Freiburg, Germany).

#### Quantification of Purkinje cell number and molecular and granular layers size

Quantification of the number of Purkinje cells was performed blindly by scanning 4 coronal sections spread over the anterior-posterior extent of the cerebellum (inter-section distance: 240 µm), using a 20× objective on a Zeiss Axiovert 200 imaging microscope and image analysis software (Image J, NIH). For each coronal section and animal, 6 fields covering the same cerebellar regions were acquired, and Purkinje cells counted in those images. For the cerebellar layers sizes, for each acquired field at least 6 measurements were made blindly in the same region for all animals, and results converted to µm using Image J software (NIH). Values are represented as the mean number of Purkinje cells per 100 µm ± SEM.

#### TUNEL assay

Cerebellar sections were stained using the *In situ* cell death detection kit, TMR Red (Roche, Mannheim, Germany) following the supplier's manual, which detects apoptotic cell death. The images were acquired digitally using a 40× objective on a Zeiss Axiovert 200 imaging microscope. All photographs for comparison were taken under identical image acquisition conditions and uniform adjustments of brightness and contrast were made to all images.

#### Fluorojade-B staining

Cerebellar sections were stained with FluoroJade-B (Chemicon, Temecula, CA), an anionic fluorescein derivative that stains neurons undergoing degeneration. The sections were mounted on glass slides, dehydrated, and stained according to the supplier's manual. The images were acquired using a Zeiss Axiovert 200 imaging microscope. All photographs for comparison were taken under identical image acquisition conditions and uniform adjustments of brightness and contrast.

#### Golgi staining

Golgi neurohistological staining was performed following a modification of classical Golgi procedure described previously [55].

## Supporting Information

File S1 **Supporting figures. Figure S1. Figure S2.**
(TIF)

File S2 **Supporting figures. Figure S3. Figure S4.**
(TIF)
