# Supplementary figures and images for: RNA Interference Mitigates Motor and Neuropathological Deficits in a Cerebellar Mouse Model of Machado-Joseph Disease
Source: PLoS One. 2014 Aug 21;9(8):e100086. doi: 10.1371/journal.pone.0100086 (PMC4140724; doi:10.1371/journal.pone.0100086)

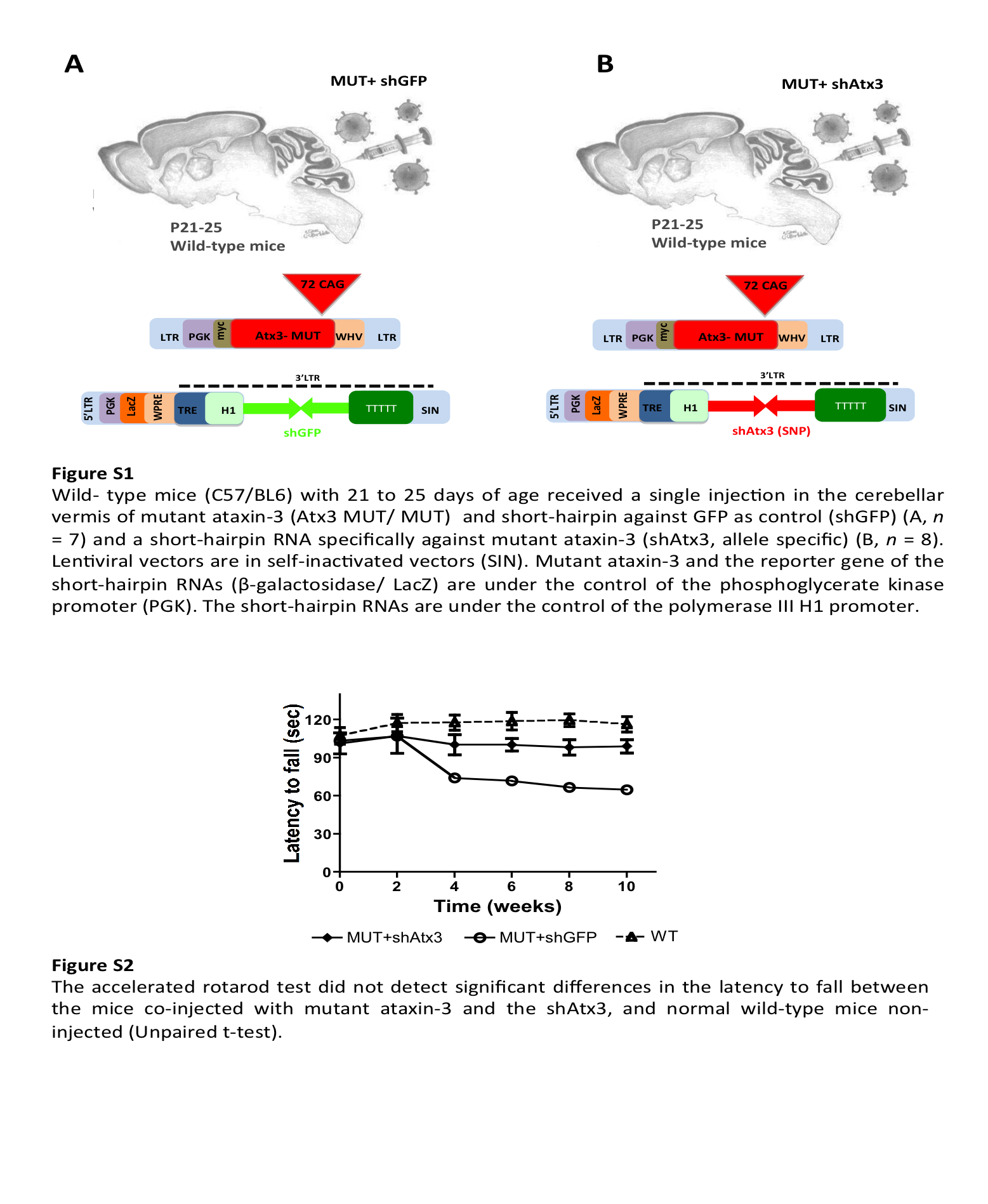

Supplement: File S1 — Supporting figures. Figure S1. Figure S2. (TIF) [file pone.0100086.s001.tif]

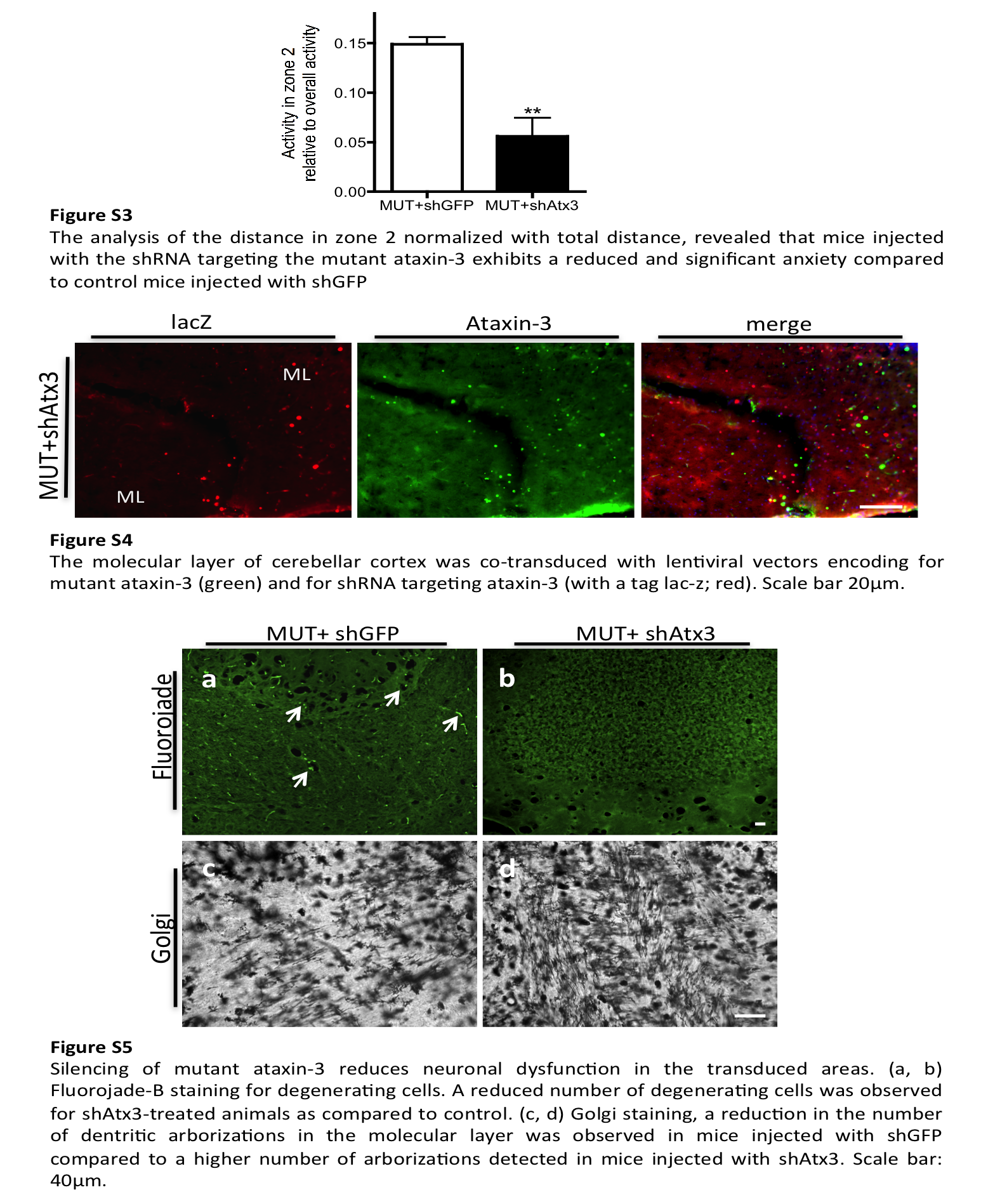

Supplement: File S2 — Supporting figures. Figure S3. Figure S4. (TIF) [file pone.0100086.s002.tif]
